# Coding-complete genome sequence of grapevine leafroll-associated virus 13 from grapevine in California

**DOI:** 10.1128/mra.00529-26

**Published:** 2026-07-30

**Authors:** Raied Abou Kubaa, Teresa M. Erickson, Haoran Li, Kristian A. Stevens, Maher Al Rwahnih

**Affiliations:** 1 Foundation Plant Services, Department of Plant Pathology, University of California 240443, Davis, California, USA; 2 Departments of Computer Science and Evolution and Ecology, University of California 8789, Davis, California, USA; 3 Department of Plant Protection, School of Agriculture, The University of Jordan, Amman, Jordan; Katholieke Universiteit Leuven, Leuven, Belgium

**Keywords:** GLRaV-13, HTS, California, grapevine

## Abstract

In this study, we report the coding-complete genome sequence of Grapevine leafroll-associated virus 13 (GLRaV-13), isolate CA8881, detected in *Vitis vinifera* in California, USA. The genome sequence exhibited over 95% nucleotide identity with previously reported GLRaV-13 isolates and contributed to better understanding of the genetic diversity of ampeloviruses infecting grapevine.

## ANNOUNCEMENT

**Fig 1:**
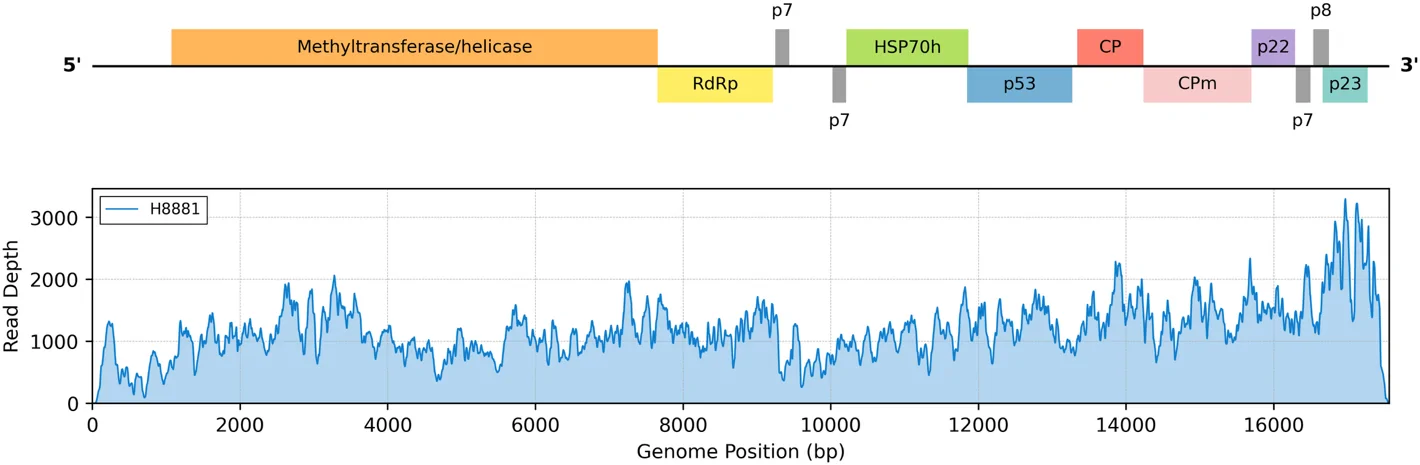
Genome organization and read depth of the Californian GLRaV-13 isolate CA8881. Coverage is continuous across the entire genome. Figure created by VirPlot (https://github.com/ucdavis/VirPlot) using NCBI Orfinder with 120 nt as the minimum ORF length to identify.

Grapevine leafroll-associated viruses (GLRaVs) are major viral pathogens of grapevine, causing significant economic losses worldwide (1). These viruses belong to the family *Closteroviridae* comprising the genera: *Closterovirus, Ampelovirus*, and *Velarivirus*. They are characterized by positive-sense single-stranded RNA (+ssRNA) genomes of ≃13–19 kb. The available genomic information about Grapevine leafroll-associated virus 13 (GLRaV-13), genus *Ampelovirus,* is still limited, with less than nine genome sequences available in public databases from Japan (2) and China (3), and some coat protein sequences from India. In this study, we report the coding-complete genome sequence of a GLRaV-13 isolate detected in *Vitis vinifera* collected in California, USA. This work expands genomic resources for GLRaV-13 and supports its detection in grapevine diagnostic programs. In February 2026, Foundation Plant Services (FPS, University of California-Davis, Davis, CA, USA) received dormant cuttings from a Southern California customer for diagnostic testing. The cuttings were collected from a vine that was exhibiting foliar leafroll symptoms. Phloem tissue was homogenized in guanidine isothiocyanate lysis buffer, and total nucleic acid was extracted using MagMAX-96 Total RNA Isolation Kit (ThermoFisher) and screened by RT-qPCR for a panel of 21 grapevine pathogens used for routine diagnostic testing at FPS. RT-qPCR (Al Rwahnih, unpublished data) revealed the presence of GLRaV-13 as well as GLRaV-3 and Grapevine virus A in the nucleic acid. The sample was then subjected to high-throughput sequencing (HTS). Briefly, the sample was ribodepleted followed by cDNA library preparation using TruSeq Stranded Total RNA with Ribo-Zero Plant Kit (Illumina, Inc., San Diego, CA). The cDNA was sequenced on an Illumina NextSeq 2000 platform, generating 23.0 million single-end reads, 100 nucleotides (nt) in length. Reads were processed as described in reference 4, consistent with current HTS-based diagnostic frameworks used in clean plant programs (5). Briefly, reads were quality-checked, adapter trimmed using bcl2fastq (v2.19; Illumina Inc), host-filtered by excluding reads that exactly align to the Grapevine genome (GCF_000003745.3) using bowtie2 v2.5.4 (6), and *de novo* assembled using SPAdes v4.2 (7), and subsequently annotated with BLASTn against GenBank. The *de novo* assembly produced a single contig of 17,564 nucleotides showing high identity to GLRaV-13 sequences in the database and confirming the RT-qPCR results. The coding-complete GLRaV-13 genome from California was assembled with an average mapped read coverage of 1,173× (Fig. 1). The 17,564 nt contig has a GC content of 45.3%, including a 5′ untranslated region (UTR) of 1,070 nt and a 3′ UTR of 223 nt. BLASTn analysis showed that the California isolate shares 95.23% nucleotide identity with GLRaV-13 reference isolate a177 (NC_029783.1) and 96.33% identity with the nearly complete genome of isolate g3-C839 (LC815114.1), both previously reported from Japan. Genome annotation using NCBI ORFfinder identified non-nested ORFs greater than 120 nt, which were annotated by BLASTp against GenBank. All ORFs associated with GLRaV-13, including replication-associated proteins (methyltransferase, helicase, RdRP), HSP70h, HSP90-like protein, coat protein (CP), minor coat protein (CPm), and several accessory proteins, were identified, reflecting the characteristic genome organization of this group. This sequence expands genomic data for GLRaV-13 and supports its detection and characterization.

## Data Availability

The complete coding genome sequence of Grapevine leafroll-associated virus 13 isolate CA8881 from California has been deposited in GenBank under the accession number PZ330031. Sequencing reads are available in the SRA under BioProject accession number PRJNA1459440.
